# Erosion-inhibiting potential of the stannous fluoride-enriched CPP-ACP complex in vitro

**DOI:** 10.1038/s41598-023-34884-4

**Published:** 2023-05-16

**Authors:** Deena Al Saady, Colin Hall, Suzanne Edwards, Eric C. Reynolds, Lindsay C. Richards, Sarbin Ranjitkar

**Affiliations:** 1 Adelaide Dental School, Level 10, Adelaide Health and Medical Sciences (AHMS) Building, University of Adelaide, Cnr George St and North Tce, Adelaide, SA 5005 Australia; 2 Future Industries Institute, University of South Australia, Mawson Lakes, Australia; 3 School of Public Health, Adelaide Health Technology Assessment (AHTA), University of Adelaide, Adelaide, Australia; 4 Oral Health Cooperative Research Centre, Melbourne Dental School, Bio21 Institute, The University of Melbourne, Melbourne, VIC Australia

**Keywords:** Diseases, Health care, Risk factors, Materials science, Nanoscience and technology

## Abstract

Currently available anti-erosive agents only provide partial protection, emphasizing the need to enhance their performance. By characterizing erosive enamel wear at the nanoscale, the aim of this in vitro study was to assess the anti-erosive effects of SnF_2_ and CPP-ACP both individually and synergistically. Erosion depths were assessed longitudinally on 40 polished human enamel specimens after 1, 5, and 10 erosion cycles. Each cycle comprised one-min erosion in citric acid (pH 3.0) and one-min treatment in whole saliva (control group) or a slurry of one of the three anti-erosive pastes (10% CPP-ACP; 0.45% SnF_2_ (1100 ppm F); or SnF_2_/CPP-ACP (10% CPP-ACP + 0.45% SnF_2_)) (n = 10 per group). Scratch depths were assessed longitudinally in separate experiments using a similar protocol after 1, 5, and 10 cycles. Compared with the control groups, all slurries reduced erosion depths after 1 cycle (*p* ≤ 0.004) and scratch depths after 5 cycles (*p* ≤ 0.012). The order of anti-erosive potential was SnF_2_/CPP-ACP > SnF_2_ > CPP-ACP > control for erosion depth analysis, and SnF_2_/CPP-ACP > (SnF_2_ = CPP-ACP) > control for scratch depth analysis. These data provide ‘proof of concept’ evidence that SnF_2_/CPP-ACP has superior anti-erosive potential compared to SnF_2_ or CPP-ACP alone.

## Introduction

Dental erosion refers to the irreversible loss of tooth structure from acids without bacterial involvement^1^. Its causes include both extrinsic factors (e.g., acidic beverages around pH of 2.5–4.0)^2,3^ and intrinsic factors (e.g., gastric regurgitation at around pH of 1.2–3.0)^4^ that can be exacerbated by mechanical wear (e.g., tooth grinding and toothbrush abrasion)^5^. Erosive wear refers to the wear process in which dental erosion is the primary aetiological factor^6^, and it is a condition of growing concern in both children and young adults because of increasing trends of acidic beverage consumption in recent years^7^. Erosive wear is cumulative over the lifetime and can result in heavily worn, debilitating dentition^8^. Therefore, clinical management should focus on early detection, prevention, stabilization, and minimally invasive approaches for aesthetic and functional rehabilitation as required^9^.

There is a consensus that surface protection plays a larger role in erosion inhibition than remineralization, given that remineralization only repairs the eroded ends of hydroxyapatite crystals rather than promoting true crystal growth^10^. Conventional fluorides, such as sodium fluoride (NaF) and amine fluoride, are commonly used in commercially available anti-erosive agents in various formulations^11^. Several alternative agents are also available in the form of calcium phosphate-based systems, polyvalent metals (e.g., Sn^2+^ and Ti^4+^), polymers (e.g., chitosan), adhesives (e.g., sealants), and protease inhibitors (e.g., chlorhexidine)^12^.

Conventional fluorides form a CaF_2_-like layer on the tooth surface and offer partial protection from dental erosion^13^, but polyvalent metal ions (e.g., Sn^2+^) have the added advantage of being incorporated into the demineralized tooth structure^14^ as well as forming a protective coating^14,15^. Fluoride alone may become insufficient in protecting teeth from demineralization under xerostomic conditions because of the low availability of Ca^2+^ and PO_4_^–3^ ions^16^. In comparison, calcium phosphate supplements, e.g., casein phosphopeptide-amorphous calcium phosphate (CPP-ACP), can replenish the depleted Ca^2+^ and PO_4_^–3^ ions^17^ in addition to forming a protective nanofilm against erosive tooth wear^18–20^. Limited evidence suggests that fluoride has a greater anti-erosive potential than calcium and phosphate^13^ and that the combination of CPP-ACP and NaF is more effective than the individual components^21^. However, currently available commercial products have limited anti-erosive potential that fall short of providing population-level benefits^22^.

Recently, SnF_2_ and CPP-ACP have been combined to form a stable paste composition that has been shown to enhance the protective effects of SnF_2_ in preventing demineralization and promoting remineralization of subsurface enamel (carious) lesions in vitro and in situ^23,24^. The inclusion of CPP-ACP with SnF_2_ was reported to enhance the uptake of Sn and F into the enamel compared with SnF_2_ alone^24^. These results suggest that SnF_2_ and CPP-ACP combined may also exhibit superior anti-erosive properties; however, to date, this has not been studied.

By using a novel technique for bioceramic wear characterization at the nanoscale, the aim of this in vitro study was to assess the anti-erosive effects of SnF_2_ and CPP-ACP both individually and synergistically at pH 3.0. The null hypothesis was that the combination of CPP-ACP with SnF_2_ would not significantly enhance protection against enamel erosion.

## Results

### Quantitative assessment

**Figure 1:**
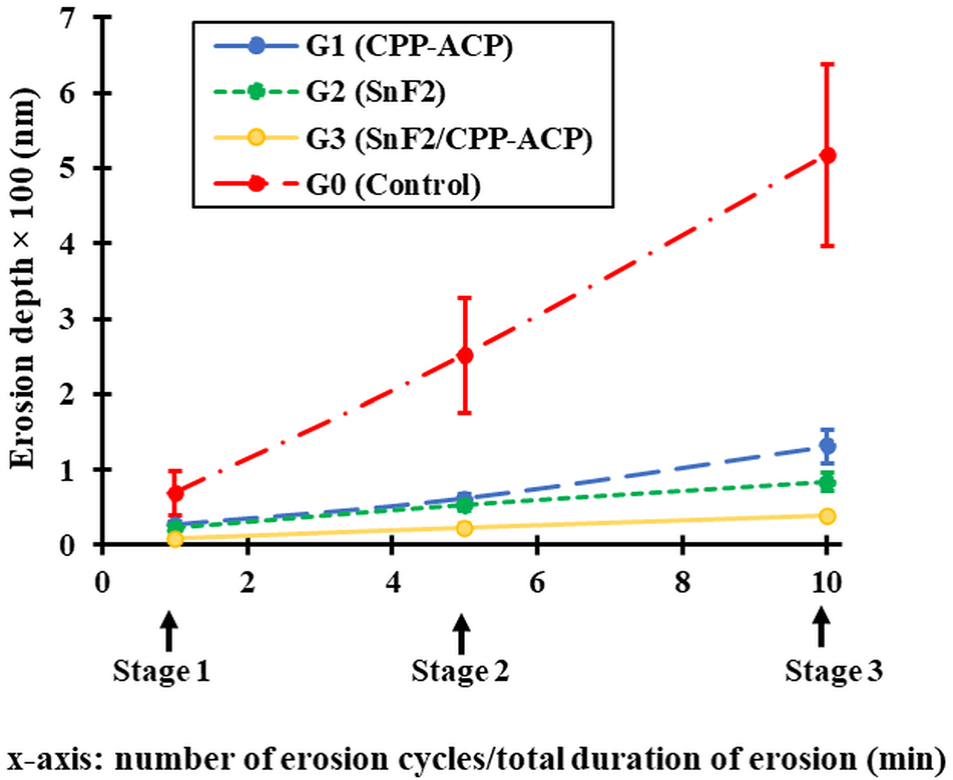
Comparison of the changes in erosion depth ± SD between the four groups from baseline to Stages 1, 2 and 3. Linear mixed-effects modeling showed significant interactions between Group and Stage (*p* < 0.001). The *p* values for pairwise comparisons are provided in Table 1.

**Figure 2:**
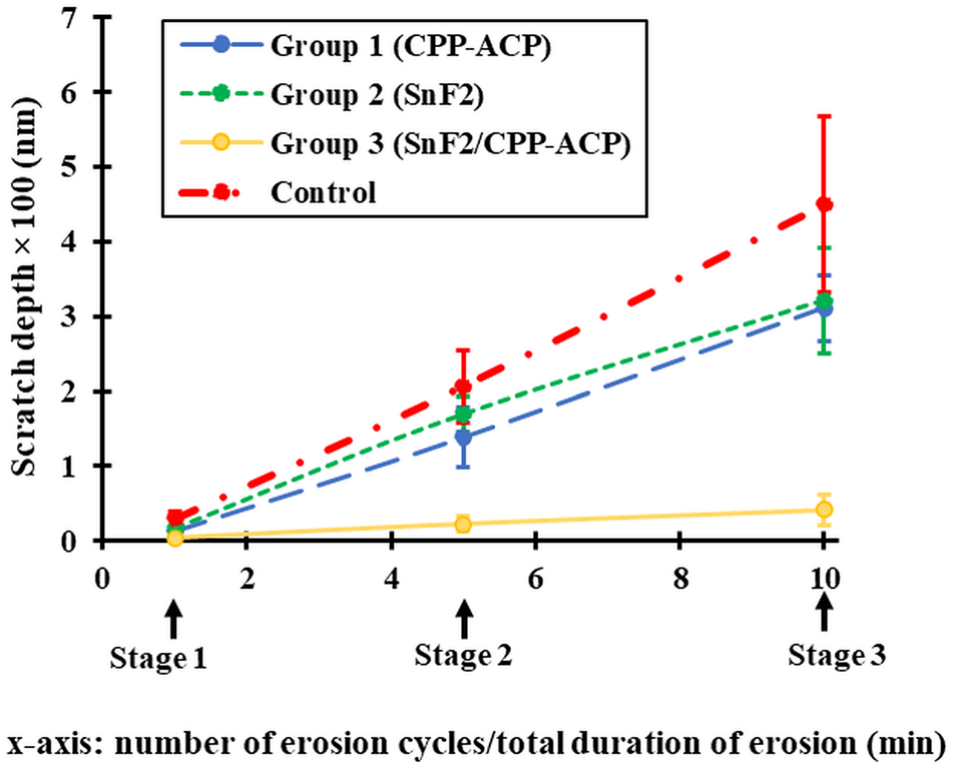
Comparison of the changes in scratch depth ± SD between the four groups from baseline to Stages 1, 2 and 3. Linear mixed-effects modeling showed significant interactions between Group and Stage (*p* < 0.001). The *p* values for pairwise comparisons are provided in Table 1.

The changes in erosion and scratch depths in different groups from baseline to Stages 1, 2 and 3 are shown in Figs. 1 and 2, and their statistical outputs are shown in Table 1. The raw data are presented in Supplementary Table S1. The mean erosion depths in the control group (Group 0) were 69.4 nm after 1 min, 252.4 nm after 5 min, and 518 nm after 10 min, and the corresponding scratch depths were 29.4 nm, 207.0 nm, and 449.8 nm, respectively.

**Table 1:** Linear mixed-effects model outputs for dependent variables ‘erosion depth’ and ‘scratch depth’ versus the interaction of Group and Stage (fixed factors), i.e., Group × Stage, adjusting for repeated measurements over time.

Parameter	Erosion depth (nm)						Scratch depth (nm)					
	Mixed-effects REML regression outputs						Mixed effects-REML regression outputs					
	No of observations = 160; no of groups = 40						No of observations = 160; no of groups = 40					
	Log restricted-likelihood = 294.54						Log restricted-likelihood = 287.99					
	Wald χ^2^ (16) = 36,522.99; *p* < 0.001						Wald χ^2^ (16) = 2220.74; *p* < 0.001					

	Coefficient	SE	z-score	*p* value	95% CI		Coefficient	SE	z-score	*p* value	95% CI	
Group												
G0	Reference group: G0						Reference group: G0					
G1	− 0.01	0.02	− 0.7	0.477	− 0.05	0.02	0.00	0.02	− 0.1	0.911	− 0.04	0.04
G2	− 0.01	0.02	− 0.4	0.729	− 0.04	0.03	− 0.03	0.03	− 1.1	0.298	− 0.08	0.02
G3	0.00	0.02	− 0.1	0.955	− 0.04	0.03	0.00	0.02	0.2	0.85	− 0.04	0.05
Stage												
S0	Reference group: S0						Reference group: S0					
S1	0.07	0.00	14.0	< 0.001	0.06	0.08	0.03	0.01	5.8	< 0.001	0.02	0.04
S2	0.25	0.01	24.5	< 0.001	0.23	0.27	0.21	0.01	19.2	< 0.001	0.19	0.23
S3	0.52	0.01	38.8	< 0.001	0.49	0.54	0.45	0.01	31.6	< 0.001	0.42	0.48
Group × Stage												
G0 × S0	Reference group: G0 × S0						Reference group: G0 × S0					
G1 × S1	− 0.04	0.01	− 6.1	< 0.001	− 0.06	− 0.03	− 0.02	0.01	− 2.2	0.027	− 0.030	− 0.002
G1 × S2	− 0.19	0.01	− 13.1	< 0.001	− 0.22	− 0.16	− 0.07	0.02	− 4.5	< 0.001	− 0.10	− 0.04
G1 × S3	− 0.39	0.02	− 20.5	< 0.001	− 0.42	− 0.35	− 0.14	0.02	− 6.9	< 0.001	− 0.18	− 0.10
G2 × S1	− 0.05	0.01	− 6.7	< 0.001	− 0.06	− 0.03	− 0.01	0.01	− 1.9	0.060	− 0.028	0.001
G2 × S2	− 0.20	0.01	− 13.7	< 0.001	− 0.23	− 0.17	− 0.04	0.02	− 2.5	0.013	− 0.07	− 0.01
G2 × S3	− 0.43	0.02	− 23.0	< 0.001	− 0.47	− 0.40	− 0.13	0.02	− 6.4	< 0.001	− 0.17	− 0.09
G3 × S1	− 0.06	0.01	− 8.7	< 0.001	− 0.08	− 0.05	− 0.03	0.01	− 3.5	0.001	− 0.04	− 0.01
G3 × S2	− 0.23	0.01	− 15.8	< 0.001	− 0.26	− 0.20	− 0.18	0.02	− 12.1	< 0.001	− 0.21	− 0.15
G3 × S3	− 0.48	0.02	− 25.4	< 0.001	− 0.52	− 0.44	− 0.41	0.02	− 20.3	< 0.001	− 0.45	− 0.37
Baseline value	1.01	0.01	174.6	< 0.001	1.00	1.03	1.64	0.35	4.7	< 0.001	0.95	2.33
Constant	− 0.02	0.02	− 1.3	0.199	− 0.05	0.01	− 0.05	0.03	− 1.6	0.119	− 0.10	0.01

Comparisons	*p* values for pairwise comparisons between different stages						*p* values for pairwise comparisons between different stages					
Stages	Group 1	Group 2	Group 3		Control		Group 1	Group 2	Group 3		Control	
S0 versus S1	< 0.001*	< 0.001*	0.105		< 0.001*		0.009*	0.002*	0.412		< 0.001*	
S0 versus S2	< 0.001*	< 0.001*	0.029*		< 0.001*		< 0.001*	< 0.001*	0.034*		< 0.001*	
S0 versus S3	< 0.001*	< 0.001*	0.004*		< 0.001*		< 0.001*	< 0.001*	0.004*		< 0.001*	
S1 versus S2	< 0.001*	0.001*	0.122		< 0.001*		< 0.001*	< 0.001*	0.056		< 0.001*	
S1 versus S3	< 0.001*	< 0.001*	0.017*		< 0.001*		< 0.001*	< 0.001*	0.006*		< 0.001*	
S2 versus S3	< 0.001*	0.003*	0.117		< 0.001*		< 0.001*	< 0.001*	0.085		< 0.001*	

Comparisons	*p* values for pairwise comparisons between different groups						*p* values for pairwise comparisons between different groups					
Groups	Baseline	Stage 1	Stage 2		Stage 3		Baseline	Stage 1	Stage 2		Stage 3	
G1 versus G0	0.477	0.003*	< 0.001*		< 0.001*		0.911	0.383	0.001*		< 0.001*	
G2 versus G0	0.729	0.004*	< 0.001*		< 0.001*		0.298	0.116	0.012*		< 0.001*	
G3 versus G0	0.955	0.001*	< 0.001*		< 0.001*		0.848	0.317	< 0.001*		< 0.001*	
G1 versus G2	0.697	0.855	0.930		0.030*		0.328	0.378	0.824		0.552	
G1 versus G3	0.508	0.765	0.159		< 0.001*		0.764	0.895	< 0.001*		< 0.001*	
G2 versus G3	0.771	0.622	0.172		0.029*		0.255	0.477	< 0.001*		< 0.001*	

The first level fixed factors (Group 0, Control group; and Stage 0, baseline) were used as ‘Reference group’ against which the effects of higher-level factors (Groups 1, 2 and 3; and Stages 1, 2 and 3) were compared.

Abbreviations: *SE* standard error of the mean, *95%CI* 95% confidence interval, *REML* Restricted Maximum Likelihood.

Restricted maximum likelihood design was used in the linear mixed-effects models instead of the unrestricted design.

*Pairwise comparisons significant at *p* < 0.05.

Treatment groups: Group 1 (erosion and CPP-ACP in a saliva slurry); Group 2 (erosion and SnF_2_ in a saliva slurry); and Group 3 (erosion and SnF_2_/CPP-ACP in a saliva slurry). Control group was subjected to erosion and saliva only (without remineralizing agent).

Stages: Stage 0 or Baseline (prior to 1st erosion cycle); Stage 1 (after the 1st erosion cycle); Stage 2 (after the 5th erosion cycle); and Stage 3 (after the 10th erosion cycle).

Linear mixed-effects modeling showed significant interactions between Group and Stage for erosion depth (*p* < 0.001), indicating that the associations between erosion depth and Group were dependent on Stage and vice versa. Post hoc pairwise comparisons for stages (baseline, S1, S2 and S3) showed the following trends: (i) *between baseline and other stages (12 comparisons):* significant increases from baseline to other stages (*p* ≤ 0.029), except one comparison of baseline vs Stage 1 in Group 3 (*p* = 0.105); and (ii) *between S1 vs S2 vs S3 (12 comparisons)*: significant increases at later stages (*p* ≤ 0.017), except two comparisons of Stage 1 versus Stage 2 (*p* = 0.122) and Stage 2 vs Stage 3 (*p* = 0.117), in Group 3. Post hoc pairwise comparisons between groups (control group vs Group 1 vs Group 2 vs Group 3) at S1, S2 and S3 stages (excluding baseline data) showed the following trends: (i) *between control and other groups (9 comparisons)*: data for Groups 1, 2 and 3 were significantly smaller than that of the control group (*p* ≤ 0.004); and (ii) *between Groups 1, 2 and 3 (9 comparisons)*: only three comparisons were significantly different at S3 (Group 3 < Group 2, *p* = 0.029; Group 3 < Group 1, *p* < 0.001; Group 2 < Group 1, *p* = 0.030). The overall trend of the anti-erosive potential of the slurries (i.e., the reverse of erosion depth progression) was Group 3 < Group 2 < Group 1 < control group. Compared with the control group, the mean erosion depths for Groups 3, 2 and 1 (after Stage 3) were smaller by 93%, 84% and 75%, respectively.

Linear mixed-effects modeling showed significant interactions between Group and Stage for scratch depth (*p* ≤ 0.027), except Group 2 × Stage 1 (*p* = 0.060), indicating that the associations between scratch depth and Group were dependent on Stage and vice versa. Post hoc pairwise comparisons for stages (baseline, S1, S2 and S3) showed the following trends: (i) *between baseline and other stages (12 comparisons):* significant increases from baseline to other stages (*p* ≤ 0.034), except one comparison of baseline vs Stage 1 in Group 3 (*p* = 0.412); and (ii) *between S1 versus S2 versus S3 (12 comparisons)*: significant increases at later stages (*p* ≤ 0.006), except two comparisons of Stage 1 versus Stage 2 (*p* = 0.056) and Stage 2 versus Stage 3 (*p* = 0.085), in Group 3. Post hoc pairwise comparisons between groups (control group vs Group 1 vs Group 2 vs Group 3) for S1, S2 and S3 stages, excluding baseline data, showed the following trends: (i) *between control and other groups (9 comparisons)*: all six comparisons for Groups 1, 2, and 3 were significantly smaller at S2 and S3 stages (*p* ≤ 0.012); (ii) *between Groups 1, 2 and 3 (9 comparisons)*: two significantly different comparisons between Group 3 versus Group 1 at S2 and S3 stages (Group 3 < Group 1, *p* < 0.001 for each comparison) and two significantly different comparisons between Group 3 vs Group 2 at S2 and S3 stages (Group 3 < Group 2, *p* < 0.001 for each comparison). The overall trend of the anti-erosive potential of the slurries (i.e., the reverse of scratch depth progression) was Group 3 < (Group 2 = Group 1) < control group. Compared with the control group, the overall mean scratch depths for Groups 3, 2 and 1 (after Stage S3) were smaller by 91%, 29%, and 31%, respectively.

### Qualitative assessment

**Figure 3:**
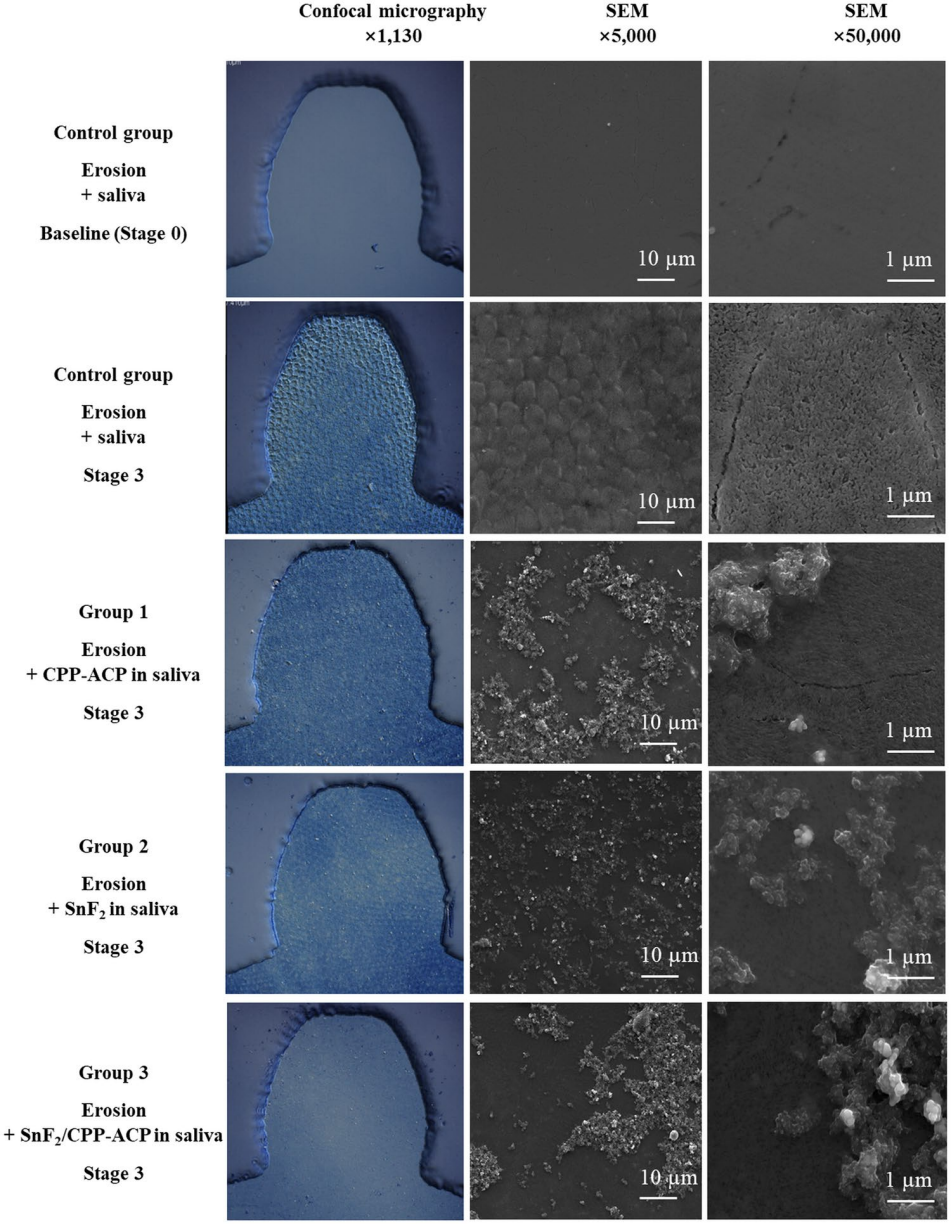
Laser confocal and scanning electron micrographic images showing the appearance of the enamel surfaces of the control group at baseline and Stage 3 and of the three other groups at Stage 3. *SEM* Scanning electron microscopy.

Figure 3 shows confocal and SEM images of flat, polished surfaces of control enamel specimens. After Stage 3, all enamel surfaces treated with anti-erosive slurries showed surface deposits in both types of micrographs. The size of these deposits observed in SEM images fell into this order: Group 2 (fine SnF_2_ particles) < Group 1 (small CPP-ACP globules) < Group 3 (larger SnF_2_/CPP-ACP agglomerates).

## Discussion

Both CPP-ACP and SnF_2_ applied alone provided protection against erosion, but they were less effective against surface softening (approximately 30% reduction in scratch depth) than against surface loss (approximately 75–80% reduction in erosion depth). In comparison, SnF_2_/CPP-ACP provided consistently higher levels of protection under both conditions. Although stannous fluoride is one of the most effective anti-erosive agents available^25^, the current data show even greater anti-erosive potential of SnF_2_/CPP-ACP (×1.3 for surface softening and ×2.0 for surface loss) (Figs. 1 and 2). These findings led to rejection of the null hypothesis that combining CPP-ACP with SnF_2_ would not significantly enhance protection against erosive enamel wear. The quantitative data were supported by both confocal and SEM images showing protective surface coatings by all three anti-erosive agents. Furthermore, the current data on the synergistic anti-erosive effects of SnF_2_ and CPP-ACP in SnF_2_/CPP-ACP are consistent with its anticariogenic potential^24^, but the anticariogenic and anti-erosive mechanisms are fundamentally different.

When CPP-ACP is combined with 900 ppm NaF, it forms casein phosphopeptide-amorphous calcium fluoride phosphate (CPP-ACFP) with a 25% greater anti-erosive potential than CPP-ACP alone^26^. In comparison, SnF_2_/CPP-ACP exceeds the anti-erosive potential of CPP-ACP by approximately 70% (current data). The key anti-erosive mechanisms of the tested slurries in the present study appear to have involved surface protection from CPP-ACP nanofilms in Group 1, Sn salts in Group 2, and Sn-peptide crosslinking in Group 3. Furthermore, the anti-erosive agents also lubricate the wear interface^27,28^, which could have partly contributed to wear reduction during scratch testing. Overall, the lowest rates of erosive wear for Sn/CPP-ACP also imply enhanced bonding of this anti-erosive paste to the enamel surfaces compared with Sn or CPP-ACP separately.

The mechanism of crosslinking between SnF_2_ and CPP-ACP and bonding with the tooth structure has been described by Fernando et al.^24^. Electron-dense Sn^2+^ binds CPP-ACFP to form a larger complex (up to 2 µm in diameter). When the Sn/CPP-ACFP complex comes into contact with the tooth surface, the positively charged Sn^2+^ and the negatively charged CPP residues adsorb to the oppositely charged ions in hydroxyapatite. Subsequently, the CPP complex undergoes a conformational change, releasing a cargo of calcium, phosphate and fluoride ions. The CPP residues also crosslink with each other to form CPP-nanofilaments. Overall, the adsorption of Sn and CPP is likely to be the main contributor to the anti-erosive properties of the SnF_2_/CPP-ACP complex, with relatively minor effects of buffering and remineralization from calcium, phosphate and fluoride release.

The present data support the notion that both surface loss and softening occur concurrently and that loss of tooth structure occurs immediately^29,30^, which differs from the view that enamel softening precedes surface loss^31^. Furthermore, the current study presents novel data for early enamel erosion, e.g., 69.4 nm in the first minute in control specimens, which could be useful for understanding the kinetics of demineralization of the hydroxyapatite crystal (width along a-axis 35.7 nm; height along c-axis 43.1 nm)^32^. The current erosion depth data of approximately 70 nm after 1 min and approximately 250 nm after 5 min are smaller than those detected by optical coherence tomography at pH 3.2 (240 nm after 1 min and 2010 nm after 5 min)^30^, but the confocal profiler has a higher resolution than optical coherence tomography.

The variation in baseline erosion data simply reflects the varying thickness of the photoresist polymer coating, ranging from 0.7 to 3.9 μm, and the variation in baseline scratch depths probably reflects the site-specific random differences in the biomechanical properties of enamel. The baseline data were adjusted during statistical analyses to improve data quality, without which the signal/noise ratio would have been too low to yield a meaningful outcome. The present approach of studying enamel erosion is also an advance over existing methods for the following reasons: (i) This is the first study demonstrating the effectiveness of anti-erosive agents in reducing erosion depth as early as 1 min (at nanoscale) compared to longer detection limits in other studies^21,33,34^, (ii) The automated calculations of erosion and scratch depths reduced operator bias, as evidenced by low technical error of blinded measurements, and (iii) The use of photolithography (nanopatterning technique) enabled longitudinal assessment of erosion depth to be made on the same spots of each specimen, removing intraspecimen variability (error) when compared to cross-sectional design in most previous studies^33,35^. Human saliva was used in the present study because of relatively large measurement errors at the nanoscale introduced by the mineral precipitates from artificial saliva. This is consistent with an earlier report that artificial saliva is not a true substitute for human saliva^36^, although it appears to be more appropriate for erosive wear testing at the microscale^37^.

The present study has the limitations of in vitro design that does not replicate the complexity of the intraoral environment. It is also possible that the physical and structural properties of the enamel were altered during sample preparation, including photolithography, although no obvious structural alteration was noted on baseline micrographs (Fig. 3). Extended periods of saliva treatment were not included between erosion cycles, in contrast to some erosive wear models simulating the complex intraoral conditions during sleep and awake stages^38,39^. While this could have overestimated the anti-erosive potential of the agents tested in the present study, a simplistic design was deemed suitable for the current ‘proof of concept’ evaluation. The promising findings now call for testing in more realistic future in vitro models, as well as appropriate in situ and in vivo models. A similar approach has been used to validate the use of surface roughness as a measure of early erosive wear in vitro^30,40^, followed by subsequent testing *in* situ^41^. Continued technological advances in 3D imaging and analytical technology^42^ hold promise to enable the development of precise in situ or in vivo models, potentially minimizing overreliance on complex in vitro models.

Future studies are also needed to determine the optimum dose and frequency of application of SnF_2_/CPP-ACP in both enamel and dentine and to better understand its protective effects under erosive conditions involving tooth grinding and toothbrush abrasion. Knowledge can be advanced by integrating various direct and indirect methods to elucidate the structural, biomechanical, and chemical (compositional) changes associated with erosive wear^43–45^. Continued effort is required to provide research thrust into translatory applications of novel materials, e.g., novel hydroxyapatite nanoparticles^46^ and calcium phosphate nanoparticles^47^, and novel techniques, e.g., sustained release of anti-erosive agents (using nanoimplants and nanoparticles)^48^, improved adhesion of the salivary biofilm^35^, electrophoresis-aided remineralization^49^, and non-thermal plasma-induced remineralization^50,51^.

## Conclusion

The current study provides ‘proof of concept’ evidence for the anti-erosive potential of SnF_2_/CPP-ACP during the early stages of erosive enamel wear. SnF_2_/CPP-ACP provided better protection against both surface loss and surface softening than CPP-ACP or SnF_2_ alone. Surface protection is likely to be the key mechanisms responsible for the anti-erosive effects of the tested materials. Overall, these findings highlight the potential role of SnF_2_/CPP-ACP in the management of enamel erosion, but further testing is required under more realistic in vitro conditions and precise in situ and in vivo situations.

## Methods

### Sample preparation

**Figure 4:**
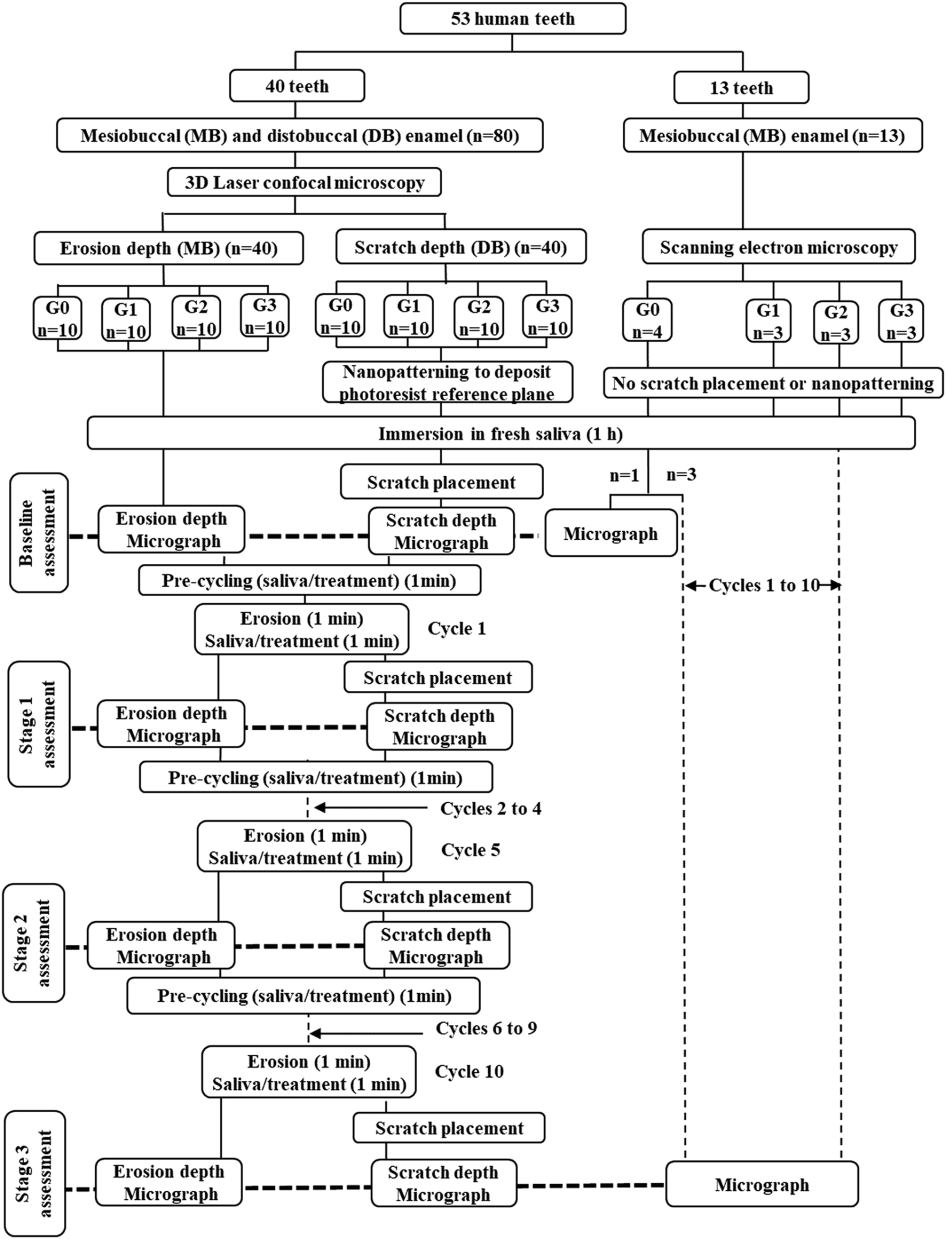
Flowchart of sampling and experimental design involving 10 erosion cycles to test the effectiveness of three anti-erosive agents in vitro. *3D* three-dimensional, *G0* control group (subjected to erosion and saliva), *G1* Group 1 (subjected to erosion and CPP-ACP in a saliva slurry), *G2* Group 2 (subjected to erosion and SnF_2_ in a saliva slurry), *G3* Group 3 (subjected to erosion and SnF_2_/CPP-ACP in a saliva slurry). Erosion was carried out with 0.3% citric acid (pH adjusted to 3.0), followed by application of anti-erosive slurry (1 g paste mixed in 20 ml saliva).

Fifty-three extracted sound human third molar teeth were collected as part of routine dental treatment in the Adelaide Dental Hospital and stored in a 0.5% thymol solution. The Human Research Ethics Committee of the University of Adelaide (H/27/90) approved the study and exempted the requirement for informed consent to collect extracted teeth. All methods were performed in accordance with the relevant guidelines and regulations. The mesiobuccal and distobuccal enamel sections from 40 teeth (n = 80 specimens) were allocated to the first part of the study, with mesiobuccal specimens (n = 40) being used for erosion testing and distobuccal specimens (n = 40) for scratch testing. The mesiobuccal enamel specimens from an additional 13 teeth (n = 13) were allocated to the second part of the study (Fig. 4). This distribution ensured that multiple specimens from the same teeth were not clustered into the same groups. All enamel sections were embedded in epoxy resin and ground flat to 0.25 µm such that approximately 2 mm × 2 mm of enamel surface was exposed in an epoxy cylinder (1.2 cm height × 1.0 cm diameter), as described previously^29^.

### Study design

A novel technique was developed to assess bioceramic wear at the nanoscale. The first part of the study involved longitudinal quantitative and qualitative assessments of erosion on 80 enamel specimens by using three-dimensional (3D) confocal microscopy (LEXT OLS5000 3D Laser Scanning Microscope; Olympus, Tokyo, Japan) with the following parameters: optical resolution X–Y 0.25 µm, Z 0.149 nm; field of view 257.678 μm × 257.410 μm; magnification × 1130. Erosive wear was quantified by measuring (i) erosion depth, a direct measure of surface loss (40 mesiobuccal specimens), and (ii) scratch depth, an indirect measure of surface softening (40 distobuccal specimens). Preliminary experiments showed that a standard artificial saliva formulation^52^ was unsuitable for nanoscale wear assessment. It caused precipitation of mineral (crystal) aggregates that could not be washed off easily, and ultrasonication only transported those deposits to new reference areas (photoresist deposits) and previously clean enamel surfaces, introducing relatively large measurement errors. In contrast, human saliva did not create such precipitates and was deemed suitable for further experimentation. The second part of the study involved a cross-sectional qualitative assessment of the eroded surfaces using scanning electron microscopy (SEM) (Quanta 450 FEG Environmental Scanning Electron Microscope; FEI Europe B.V., Eindhoven, the Netherlands) on the remaining mesiobuccal specimens (n = 13) (Fig. 4).

Only one specimen was processed at a time from start to finish rather than processing multiple specimens in the same container. Prior to experimentation, all specimens were cleaned in an ultrasonic water bath for 10 min and then treated with human whole saliva for 1 h. Stimulated saliva was collected from a healthy donor after 2-h of fasting at 09.00 h and 12.00 h, and anti-erosive slurries were prepared and used within 3 h of saliva collection. The salivary parameters that were collected using a saliva kit (GC Saliva-Check Buffer Kit; GC Corporation, Tokyo, Japan) on 20 occasions over a 10-month period were pH 6.8–7.8; stimulated saliva flow rate > 5.0 ml at 5 min; and buffering capacity 10–12. The anti-erosive slurries were prepared for the experimental groups by mixing one of the three anti-erosive agents in whole saliva (1 g per 20 ml): (i) Group 1 (CPP-ACP): 10% CPP-ACP (Tooth Mousse; GC Corporation, Tokyo, Japan); (ii) Group 2 (SnF_2_): 0.45% SnF_2_ (3440 ppm Sn + 1100 ppm fluoride) (Test product 1; GC Corporation, Tokyo, Japan); and (iii) Group 3 (SnF_2_/CPP-ACP): 0.45% SnF_2_ (3440 ppm Sn + 1100 ppm fluoride) enriched 10% CPP-ACP (Test product 2; GC Corporation, Tokyo, Japan). The final concentrations of anti-erosive slurries were 0.5% CPP-ACP and 0.0225% (555 ppm) SnF_2_ (420 ppm Sn and 135 ppm F). Only whole saliva (without anti-erosive agent) was applied in the control group.

In the first part of the study, enamel specimens were assigned randomly to 3 experimental groups (Groups 1 to 3) and a control group (n = 10 per group). All specimens were subjected to 10 erosion cycles, with each cycle comprising 1 min erosion (0.3% citric acid, pH adjusted to 3.0) and 1 min treatment in whole saliva or the anti-erosive slurry. Erosion was carried out by applying 0.5 ml of citric acid to wash off the enamel surface, and then by leaving it undisturbed for the remainder of the erosion phase. Then, 0.5 ml of whole saliva was applied on the enamel surface for 2 s to rinse off citric acid, and copious amounts of saliva were used to wash off the wall and the base of each epoxy disc. The treatment phase of the erosion cycle was carried out by immersing the whole epoxy discs into 20 ml of whole saliva or anti-erosive slurry. The quantitative and qualitative assessments were performed at 4 stages: baseline (Stage 0; before the 1st erosion cycle) and at the end of the 1st, 5th and 10th erosion cycles (Stages 1, 2 and 3, respectively) corresponding to cumulative erosion for 1 min, 5 min and 10 min, respectively. At Stages 1, 2 and 3, the erosion cycles were stopped, and the specimens were gently dried with air for 5 s before erosion and scratch depths were assessed at room temperature (approximately 40 min per stage). At the completion of assessments at baseline, Stage 1, and Stage 2, the specimens were subjected to precycling with saliva or anti-erosive slurry for 1 min before the next erosion cycle began.

**Figure 5:**
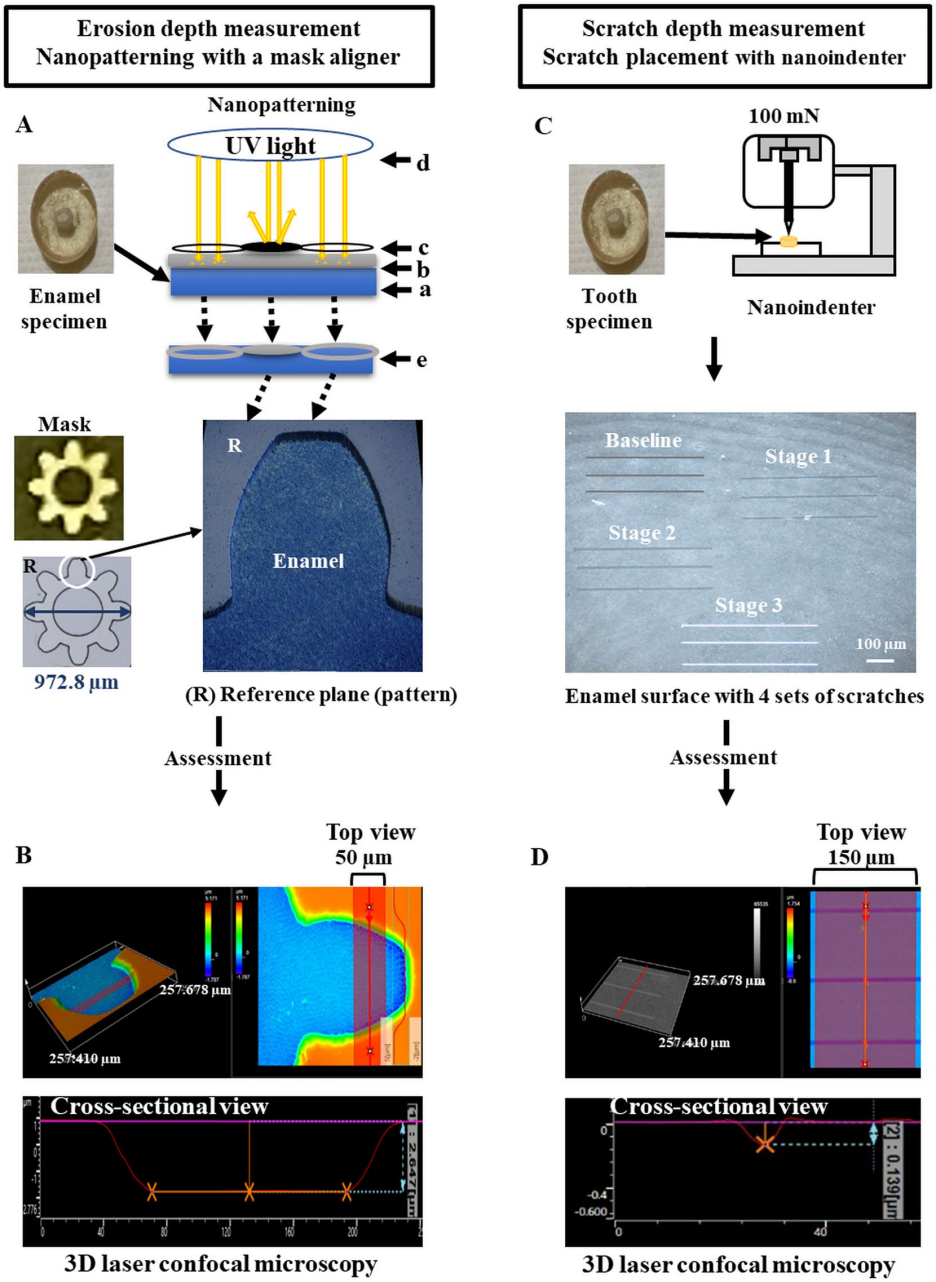
Scheme of the enamel erosion experiments involved in measuring (**A**, **B**) erosion depth and (**C**, **D**) scratch depth by using 3D laser confocal microscopy. (**A**) Photolithography (nanopatterning technique) was used to deposit an external reference resin layer on (a) flat, polished enamel surface by (b) spin-coating a layer of ‘positive’ photoresist resin/polymer (AZ 1518) (followed by heating at approximately 38 °C for 5 min), (c) placing a photolithographic mask (transparent soda-lime glass with areas of opaque chrome coating) with 8 U-shaped arms on the resin, and (d) exposing the resin to ultraviolet light for 5 s, which disintegrated the polymer layer that was not covered by the chrome (and the disintegrated polymer was subsequently removed by treating with an alkaline developer for 30 s, washing with water for 30 s and gently drying with air). This produced a positive pattern on the enamel surface with (e) coated regions (with photoresist resin) or uncoated regions (exposed enamel where the resin layer was removed). (**B**) 3D images (field of view 257.678 µm × 257.410 µm) were acquired to calculate erosion depth across a 50-µm length (top view shows a U-shaped arm and cross-sectional view shows erosion depth measured from the photoresist reference plane). (**C**) Flat, polished enamel specimens were subjected to scratch placement (3 sets of 500-µm long scratches at 4 different stages) in a nanoindenter using a 20 µm radius tip under a load of 100 mN. (**D**) 3D images (field of view 257.678 µm × 257.410 µm) were acquired to calculate scratch depth across a 150-µm length (top view shows 3 horizontal scratches in dark purple color, and cross-sectional view shows scratch depth measured from the enamel edges).

For erosion depth analysis, photolithography was used for deposition (microfabrication) of acid-resistant ‘photoresist’ polymer (resin) on the enamel surface. This deposit was used as a reference plane for longitudinal assessment of erosion on the same surface in subsequent stages. Photolithography is commonly used for surface patterning in microelectronic fabrication by selectively etching a thin film or silicon wafer to create a specific geometric pattern^53,54^. A pattern with 8 U-shaped arms (Fig. 5) was formed on the enamel surface via a 4-step process: (i) spin-coating a thin, uniform layer of ‘positive’ photoresist resin (Photoresist AZ 1518; Telic Company, Valencia, CA, USA) on the enamel surface, (ii) heating the base of the epoxy disc with a hot plate at 100 °C for 5 min (approximately 38 °C on the exposed enamel surface) to promote adhesion, (iii) applying a photolithographic mask (transparent ‘soda-lime glass’ covered with areas of opaque chrome coating) on the resin before exposing it to ultraviolet light for 5 s, after which the exposed polymer disintegrated and the unexposed polymer (covered by the chrome) remained intact, and (iv) removing the disintegrated resin by applying an alkaline developer (AZ 726 MIF; AZ Electronic Materials USA Corp.70, Somerville, NJ, USA) for 30 s and washing with water for 30 s, followed by gentle air-drying. The entire process resulted in the deposition of a photoresist resin reference plane with a thickness of 0.7 to 3.9 µm.

Nanoscratches were placed with a 20-µm radius spherical diamond tip in a nanoindenter (Ultra-Micro Indentation System – 2000; The Commonwealth Scientific and Industrial Research Organisation (CSIRO), Sydney, Australia) under a load of 100 mN (Fig. 5). Nanoscratch testing is a valid and essential component of studying the tribological (wear) behavior of both thin coatings and bulk materials^55,56^, including tooth enamel^45,57–59^. The scratch parameters for erosive wear testing were adopted from a previous study, as they provided an adequate signal-to-noise ratio for the detection of enamel softening without bulk failure^60^. The microscope optics attached to the nanoindenter helped to locate the correct areas for scratch placement. Four sets of three parallel 500-µm-long nano-scratches were placed on the enamel surface at least 400 µm away from the epoxy border and away from obvious cracks or defects. Specifically, these scratches were placed on the top-left corner at baseline, the middle-right region at Stage 1, the middle-left region at Stage 2, and the bottom-right region at Stage 3. The scratches were separated at least 50 μm apart to prevent microcracks and plastic deformation from one scratch affecting others.

For the second part of the study involving SEM analysis, the same experimental protocol for erosion testing was used but without depositing the photoresist resin reference plane. The 13 specimens were divided into 4 groups: Group 1 (n = 3), Group 2 (n = 3), Group 3 (n = 3) and the control group (n = 4). One control specimen was subjected to SEM analysis at baseline, and the rest (n = 12) were subjected to 10 erosion cycles.

### Assessment of erosive wear

Changes in both scratch and erosion depths were assessed from baseline to Stages 1, 2 and 3 using 3D topographical analysis software (LEXT OLS 5100; Olympus, Tokyo, Japan) (Fig. 5). All scans were corrected for tilt prior to analysis. The erosion depth was measured along the upper region of the U-shaped arm, so that the middle of the scanned pitch (approximately 50 µm long) was separated by at least 30 µm from the photoresist border. The same surface was located for longitudinal assessment at subsequent stages with the aid of microscope optics. Erosion depth was calculated from the reference plane (photoresist resin) to the enamel surface by averaging all the vertical distances (spaced at 0.25 µm) across the entire scanned pitch. The scratch depth was measured only on freshly placed scratches towards the middle-third (150 µm) of the scanned pitch. Older scratches placed from earlier stages were not used for analysis. Scratch depth was calculated by averaging the vertical distances from the reference plane fitted over the enamel edges onto the body of the scratch (spaced at 0.25 µm) across the 150-µm long pitch. The data for the 3 scratches were averaged to determine the mean scratch depth for each specimen at each stage. For SEM analysis, the specimens were carbon-coated after being air-dried and dehydrated with silica gel for 72 h.

### Statistical analysis

Statistical analysis was performed by using a statistical package (Stata 15.1; StataCorp; College Station, TX, USA). Power calculations showed that a sample size of 9 to 10 specimens was required in each group, assuming an effect size of 0.6, type I error probability (α) of 0.05, and power (1-β) of 0.80 for comparison of mean values between the four groups displaying intermediate dispersion of individual values. This conforms to previous studies conducted using similar testing methods^29,60^. Linear mixed-effects regression models were performed to determine whether the mean erosion depths and scratch depths (dependent variables) were significantly different between Group and Stage (fixed factors). The analyses were controlled for random errors from individual specimens and adjusted for baseline erosion and scratch depths (confounders). Significance was set at the 0.05 level.

### Error testing

The method of double determination was used to test for systematic and random errors when calculating erosion and scratch depths at baseline and Stage 3 in 24 randomly selected scans (4 groups × 3 specimens per group × 2 stages for each parameter). The same examiner (DL) collected the data under blinded conditions 2-weeks apart. Paired t-tests showed no significant systematic errors between the repeated measurements (*erosion depth*, n = 24; t statistic =  − 1.09, degrees of freedom = 23, *p* = 0.285; *scratch depth*, n = 24; t statistic =  − 0.17, degrees of freedom = 23, *p* = 0.866), and Dahlberg’s technical error of measurements was low (< 3%).

## Supplementary Information

Supplementary Information.

## Data Availability

The datasets generated and/or analysed during the current study are available from the corresponding author on reasonable request.
