# Supplementary material for: Erosion-inhibiting potential of the stannous fluoride-enriched CPP-ACP complex in vitro
Source: Sci Rep. 2023 May 16;13:7940. doi: 10.1038/s41598-023-34884-4 (PMC10188480; doi:10.1038/s41598-023-34884-4)
Supplement: Supplementary file 1 — Supplementary Information. [file 41598_2023_34884_MOESM1_ESM.pdf]

Supplementary Information for

Erosion-inhibiting potential of stannous fluoride-enriched CPP-ACP complex *in vitro*

Deena Al Saady[1], Colin Hall[2], Suzanne Edwards[3], Eric C. Reynolds[4], Lindsay C. Richards[1], Sarbin Ranjitkar[1,*]

[1]Adelaide Dental School, University of Adelaide, Adelaide, Australia

[*]Email: sarbin.ranjitkar@adelaide.edu.au

[2]Future Industries Institute, University of South Australia, Mawson Lakes, Australia

[3]Adelaide Health Technology Assessment (AHTA), School of Public Health, University of Adelaide, Adelaide, Australia

[4]Oral Health Cooperative Research Centre, Melbourne Dental School, Bio21 Institute, The University of Melbourne, Melbourne, Victoria, Australia

Group	Baseline (S0)		Stage 1 (S1)		Stage 2 (S2)		Stage 3 (S3)	
	Mean	SD	Mean	SD	Mean	SD	Mean	SD
Erosion depth								
G1	2395.5	1089.7	2421.8	1091.8	2457.3	1091.5	2526.6	1104.7
G2	1877.0	1086.5	1899.3	1088.8	1930.0	1092.3	1960.8	1097.3
G3	1491.2	810.6	1499.3	810.2	1513.7	810.7	1530.0	810.9
G0	1416.4	945.2	1485.8	964.3	1668.8	995.7	1934.4	1032.0
Scratch depth								
G1	76.5	14.4	89.7	16.2	215.3	49.6	387.7	54.1
G2	115.0	30.8	130.8	29.5	283.8	47.5	435.9	91.8
G3	66.2	12.0	70.5	11.6	89.1	14.1	107.6	23.3
G0	72.6	14.9	102.0	15.6	279.6	50.6	522.4	121.5

Abbreviations: SD = standard deviation; G1 (Group 1) = Erosion + CPP-ACP in saliva; G2 (Group 2) = Erosion + SnF_2 in saliva; G3 (Group 3) = Erosion + SnF_2/CPP-ACP in saliva); G0 (Control group) = Erosion + saliva

Table S1. Erosion depth (nm) and scratch depth (nm) data at baseline (S0) and subsequent stages (S1, 1 min; S2, 5 min; and S3, 10 min of erosion) that were analyzed by using linear mixed-effects models, controlling for baseline. The changes from baseline for erosion depth are shown in Fig. 1, and for scratch depth in Fig. 2.
